# Potential plasma markers of type 1 and type 2 leprosy reactions: a preliminary report

**DOI:** 10.1186/1471-2334-9-75

**Published:** 2009-05-27

**Authors:** Mariane M Stefani, Jackeline G Guerra, Ana Lucia M Sousa, Mauricio B Costa, Maria Leide W Oliveira, Celina T Martelli, David M Scollard

**Affiliations:** 1 Tropical Pathology and Public Health Institute, Federal University of Goias, Goiania, GO, Brazil; 2 Faculty of Medicine, Federal University of Goias, Goiania, GO, Brazil; 3 Federal University of Rio de Janeiro, Rio de Janeiro, RJ, Brazil; 4 National Hansen's Disease Programs, Baton Rouge, LA, USA

## Abstract

**Background:**

The clinical management of leprosy Type 1 (T1R) and Type 2 (T2R) reactions pose challenges mainly because they can cause severe nerve injury and disability. No laboratory test or marker is available for the diagnosis or prognosis of leprosy reactions. This study simultaneously screened plasma factors to identify circulating biomarkers associated with leprosy T1R and T2R among patients recruited in Goiania, Central Brazil.

**Methods:**

A nested case-control study evaluated T1R (n = 10) and TR2 (n = 10) compared to leprosy patients without reactions (n = 29), matched by sex and age-group (+/- 5 years) and histopathological classification. Multiplex bead based technique provided profiles of 27 plasma factors including 16 pro inflammatory cytokines: tumor necrosis factor-α (TNF-α), Interferon-γ (IFN-γ), interleukin (IL)- IL12p70, IL2, IL17, IL1 β, IL6, IL15, IL5, IL8, macrophage inflammatory protein (MIP)-1 alpha (MIP1α), 1 beta (MIP1β), regulated upon activation normal T-cell expressed and secreted (RANTES), monocyte chemoattractrant protein 1 (MCP1), CC-chemokine 11 (CCL11/Eotaxin), CXC-chemokine 10 (CXCL10/IP10); 4 anti inflammatory interleukins: IL4, IL10, IL13, IL1Rα and 7 growth factors: IL7, IL9, granulocyte-colony stimulating factor (G-CSF), granulocyte macrophage-colony stimulating factor (GM-CSF), platelet-derived growth factor BB (PDGF BB), basic fibroblast growth factor (bFGF), vascular endothelial growth factor (VEGF).

**Results:**

Elevations of plasma CXCL10 (P = 0.004) and IL6 (p = 0.013) were observed in T1R patients compared to controls without reaction. IL6 (p = 0.05), IL7 (p = 0.039), and PDGF-BB (p = 0.041) were elevated in T2R. RANTES and GMCSF were excluded due to values above and below detection limit respectively in all samples.

**Conclusion:**

Potential biomarkers of T1R identified were CXCL10 and IL6 whereas IL7, PDGF-BB and IL6, may be laboratory markers of TR2. Additional studies on these biomarkers may help understand the immunopathologic mechanisms of leprosy reactions and indicate their usefulness for the diagnosis and for the clinical management of these events.

## Background

Leprosy reactions are major causes of hospitalization and disability of patients with leprosy, a chronic granulomatous disease of the skin and peripheral nerves caused by the *Mycobacterium leprae*. These are understood to be consequences of the dynamic nature of the immune response to *M. leprae*, and they may occur before diagnosis, during treatment or after multidrug therapy (MDT). Leprosy "Type 1" reactions (T1R), also described as "reversal" reactions, occur in 30–40% of borderline patients with cellular immune responses to *M. leprae *[1]. T1R usually develops abruptly as exacerbations of pre-existing skin and nerve lesions. Treatment with corticosteroids alleviates symptoms, but often requires many weeks or months of moderate to high doses.

"Type 2" reactions (T2R), also known as *erythema nodosum leprosum *(ENL), occur only in lepromatous (LL) and borderline lepromatous (BL) patients with a high bacterial load and little or no cellular immunity to *M. leprae *[1]. T2R develop abruptly, with crops of red, tender nodules on various parts of the body, and histological evidence of acute inflammation. The natural course of T2R is typically 10 – 14 days, but without treatment severe tissue damage, including nerves, often results. Corticosteroids and thalidomide reduce the inflammation in T2R, but many patients have multiple, recurrent episodes.

Neuritis and cutaneous inflammation are prominent symptoms of both types of reaction, but the systemic nature of these reactions is evident from the patterns of malaise, fever, and inflammation in other tissues that are characteristic of both T1R and especially T2R reactions. In leprosy reactions, peripheral immunological and inflammatory mechanisms are undoubtedly important, but enhanced central immunological reactivity in spleen and lymph nodes may contribute to the overall illness. In human studies, plasma provides an appropriate sample of the soluble mediators produced by central lymphoid organs.

Cytokines play important roles in both protection and immunopathology of leprosy and are considered important components of leprosy reactions: reviewed in [2]. T1R have been characterized by in situ Th1 type immunity with spontaneous increase in cell mediated immunity with infiltration of IFNγ and TNFα, secreting CD4^+^T cells in skin lesions and nerves. The T2R is a systemic inflammatory response characterized by neutrophil infiltration, activation of complement, extra-vascular immune complexes and high levels of TNFα in tissue lesions and in the circulation [3-5]. The presence of *M. leprae *DNA detected by PCR in skin lesions has been demonstrated to be a molecular marker of T1R among single skin lesion PB leprosy [6]. Despite evidences of immunological and molecular mediators of leprosy reactions, no individual marker or combination of markers has been sufficiently associated with wither reaction to enable its use as a laboratory test for the diagnosis or management of T1R or T2R.

The goal of this study was to screen potential plasma markers in Type 1 and Type 2 leprosy reactions. Profiles of 27 markers including 16 circulating pro inflammatory, 4 anti inflammatory cytokines and 7 growth factors were compared among T1R and T2R patients and leprosy patients without reaction (matched for age, sex, and leprosy type).

## Methods

### Patients

This study was designed as a nested case-control study within a cohort of 349 newly detected untreated leprosy patients recruited between February 2004 and October 2005 at a main outpatient clinic in Central Brazil (Reference Center for Diagnosis and Treatment, Goiania city). Eligibility criteria were: newly diagnosed untreated leprosy patients. Leprosy patients living outside the study area or patients not willing to come for follow up were excluded. At baseline, patients were given complete dermatological and neurological evaluations by a dermatologist and a physiotherapist with expertise in leprosy diagnosis. Slit skin smears and skin lesion biopsies were collected at baseline and histhopathological readings were classified according to Ridley-Jopling criteria [7]. All patients received the standard WHO MDT regimen for PB or MB disease and patients were followed-up during MDT to monitor any episode of leprosy reaction. Leprosy reactions were treated according to the guidelines from the Brazilian Leprosy Control Program.

In this study, T1R cases were selected from untreated leprosy patients clinically diagnosed with T1R (n = 10) the time of their initial diagnosis. These patients had severely indurated and erythematous lesions at the site of previously indolent macules, according to the medical history. T2R cases (n = 10) were selected among patients who had the T2R at diagnosis (n = 4) or during follow up (n = 6). The following case definitions were employed: T1R was defined as an acute clinical manifestation, usually characterized by the exacerbation of pre-existing lesions, the appearance of new lesions with or without neuritis. T2R was characterized by the sudden appearance of tender erythematous skin nodules mainly accompanied by fever and other systemic symptoms such as joint pain, bone tenderness, neuritis, edema, malaise, anorexia with or without lymphadenopathy. Controls were selected among leprosy patients with the same histopathological classification as the reaction patients but did not have reaction at the time of initial diagnosis or during follow-up. These controls were also matched by sex and age-group (+/- 5 years). Ten controls were found suitable for TR1 and 9 controls for TR2.

### Data Collection and Ethical Issues

A standardized Case Report Form (CRF) was applied to all patients to collect data on age, sex, duration of symptoms, clinical characteristics, neurological impairment, onset of reaction and final classification. This study was approved by the institutional review board and all patients gave written, informed consent.

### Blood Collection and Cytokine Analysis

Blood was collected in EDTA, centrifuged immediately, and plasma aliquots were stored at -80°C prior to assay.

### Cytokine Array System

This study employed a commercially available premixed human cytokine 27-plex panel of cytokines (Bio-Plex Cytokine reagent kit, BIO RAD Laboratories, Hercules, CA, USA). This system consists of multiplex beads and detection antibodies designed to quantitate, in pg/ml, multiple cytokines in plasma samples. This study employed the premixed 27-plex panel of human cytokines. For data analysis the 27 cytokines were stratified into 3 functional categories: A. Pro-inflammatory cytokines- TNFα, IFNγ, IL12, IL2, IL17, IL1 β, IL6, IL15, IL5, IL8, CXCL10, MIP1α, MIP1β, RANTES, MCP1 and CCL11; B. Anti- inflammatory cytokines- IL4, IL10, IL13 and IL1Rα; C. Growth Factors- IL7, IL9, GMCSF, GCSF, PDGF-BB, FGF-Bas and VEGF.

EDTA plasma samples stored at -80°C were thawed, centrifuged at 1,000 × g at 4°C for 10 min, supernatants were filtered (sterile 0.22 um Millipore filters) and used immediately. All samples were tested in duplicate at 1/3 dilution in species specific Bio-plex sample diluent, and blank values (background) were subtracted from all readings. Premixed lyophilized cytokine standards, premixed anti cytokine conjugated beads, detection antibodies and Streptavidin-PE were prepared according to manufacturer instructions. All assays were read on the Luminex 100 Instrument (Austin, Texas) and data analysis performed with the Bio-Plex-Manager software which includes determination of assay precision and determination of goodness of fit of the regression algorithm.

### Statistical Analysis

The concentration of individual cytokines in pg/ml in each patient was used for data analysis. Descriptive statistics were applied to the patient's characteristics. Exploratory data analysis, including box-plot, medians and standard deviation were calculated for the concentration of each cytokine and results were stratified by groups of cases (T1R and T2R) and compared to plasma concentrations obtained in their respective control groups. Statistical significance was assessed by Kruskall-Wallis one way analysis of variance for comparison of multiple groups and Mann-Whitney for comparison between two groups. P values < 0.05 were considered statistical significant results.

## Results

### Patients' characteristics

The main baseline clinical characteristics of the 39 leprosy patients tested during T1R (n = 10) and controls (n = 10) or T2R (n = 10) and controls (n = 9) are shown in Table 1. Adult males around 40 years of age predominated in all groups. Patients with T1R and controls were classified by clinical and histopathological criteria as BT and none had acid-fast bacilli in skin smears (BI = 0). Patients with T2R and controls had LL disease; all had acid-fast bacilli in skin smears. For patients diagnosed during a reactional episode (10/10 T1R and 4/10 T2R cases), a wide variation in the duration of symptoms prior to diagnosis was reported: median of 50 days for T1R and 15 days for T2R. No clinical differences were identified between patients who had T2R on diagnosis compared to those who developed the reaction after starting MDT. None of the leprosy patients included in the study had neuritis. Prednisolone was the treatment of choice for all T1R patients. T2R patients were treated with steroid (n = 6) or thalidomide (n = 3) or steroid and thalidomide combination (n = 1).

**Table 1:** Baseline clinical characteristics of patients with leprosy reactions and controls without reaction

Characteristics	T1R^1^	Controls^2^	T2R^3^	Controls^4^
Patients n (%)	10	10	10	9
Sex				
men	8 (80.0)	8 (80.0)	8 (80.0)	7 (77.8)
women	2 (20.0)	2 (20.0)	2 (20.0)	2 (20.0)
Age (median)	42.9	41.4	38.9	43.3
(min-max)	(26–60)	(26–59)	(17–69)	(13–68)
Classification				
BT	10	10	-	-
LL	-	-	10	9
Baciloscopic Index				
(median)	0	0	3.25	2.75
(min-max)	(-)	(-)	(2.5–5.0)	(2.5–5.0)
Reaction at diagnosis	10 (100.0)	-	04 (40.0)	-
Duration of symptoms prior to diagnosis (days)				
(median)	50	-	15	-
(min-max)	(3–120)		(3–60)	

^1 ^Type 1 Reaction

^2 ^Paucibacillary leprosy patients without reaction

^3 ^Type 2 Reaction

^4 ^Multibacillary leprosy patients without reaction

### Cytokine Analysis

Regardless of the study group and cytokine classification, a wide variation was observed in the plasma concentration of cytokines tested (data not shown). Among pro-inflammatory cytokines, IL-6 levels ranged from 31.81 to 724 pg/ml, median = 103.20 (SD = 129.42). For anti-inflammatory cytokines IL1Rα values ranged from 44.24 pg/ml to 2642 pg/ml, median = 179.52 (SD = 529.63). Among growth factors the widest variation was observed fro PDGF BB concentration (median = 4264.47; SD = 3131.78). Two out of 27 cytokines tested, RANTES and GMCSF were excluded from the analysis because their values were above and below detection limit respectively. High RANTES levels were probably artifacts, since platelets had not been removed from the plasma prior to freezing.

### Pro-inflammatory cytokines

**Figure 1:**
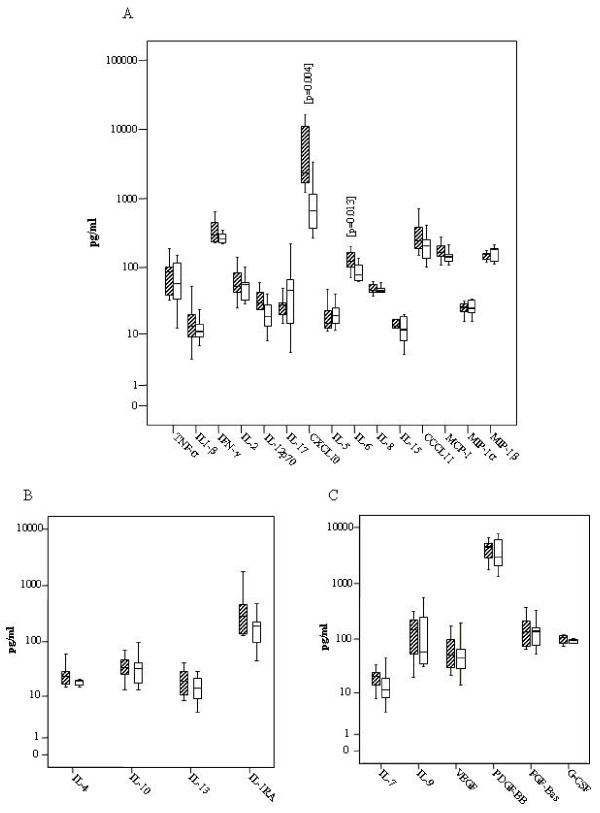
**Distribution of cytokines among Type 1 reaction leprosy patients (traced boxes) and controls without reaction (white boxes)**. (A) Pro inflammatory Cytokines, CXCL10 and IL-6 median values were statistically higher for TR1 than controls (B) Anti Inflammatory cytokines (C) Growth Factors. Boxes encompass 25th and 75th percentiles of each cytokine distribution. Black lines within boxes refer to the median values.

**Figure 2:**
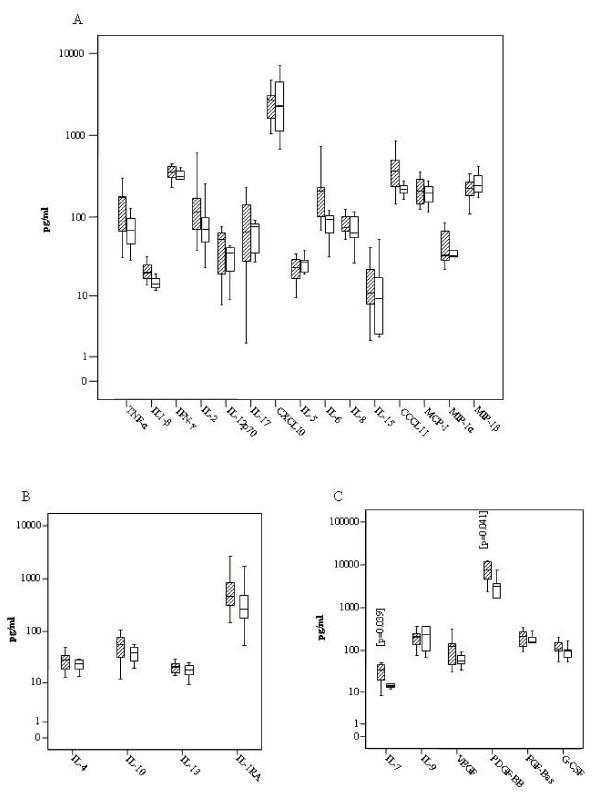
**Distribution of cytokines among Type 2 reaction leprosy patients (traced boxes) and controls without reaction (white boxes)**. (A) Pro inflammatory Cytokines (B) Anti Inflammatory cytokines (C) Growth Factors, IL-7 and PDGF-BB median values were statistically higher among cases than in the control group. Boxes encompass 25th and 75th percentiles of each cytokine distribution. Black lines within boxes refer to the median values.

Box plots distributions indicate differences in median plasma concentrations for all of the 15 inflammatory cytokines stratified by T1R cases and controls (Figure 1A) and T2R and controls (Figure 2A). The difference of CXCL10 median plasma concentration among T1R and their matched controls was highly significant (p = 0.004) (Fig 1A). IL6 also showed statistically significant differences between T1R patients and matched controls (p = 0.013) (Fig. 1A), while the difference for T2R patients and matched controls did not reach statistical significance (P = 0.05) (Fig. 2A). The differences between medians of CCL11 concentrations among T2R (363.6 pg/ml) and controls (218.6 pg/ml) were notable but not statistically significant (p = 0.06) (Fig. 2A).

The differences between median values of TNFα, IFN γ, IL1 β, IL12, IL15, IL2, IL5, IL8, MIP1α, MIP1β, IL17, MCP1 were not statistically different (p > 0.05) for non-reactional T1R-controls and T2R-controls (Figures 1A and 2A). For TNFα the median for T2R cases was 171.8 pg/ml and for the control group without reaction the median was 67.7 pg/ml (p = 0.297). The medians of IFNγ for T2R and controls were 358.0 pg/ml and 315.4 pg/ml respectively (Fig. 2A). No associations were observed between cytokine and chemokine concentrations and the duration of reaction symptoms (data not shown), although the sample may be too small to reliably determine this.

### Anti-Inflammatory cytokines

The difference between median values of IL10 plasma concentrations among T2R patients (median = 56.9 pg/ml) and controls (median = 39.4 pg/ml) was marginally significant (p = 0.07) (Figure 2B). Regarding IL4, IL13 and IL1Rα there was no statistically significant difference in the plasma concentration medians between type 1 and type 2 reactional groups and their respective control leprosy groups without reaction (Figures 1B, 2B).

### Growth Factors

Regarding IL7, IL9, GMCSF, GCSF, PDGF-BB, FGF-Bas and VEGF, no difference was observed among T1R and controls (Figure 1C). For T2R the difference of medians between cases and controls was statistically significant for IL-7 (p = 0.039) and PDGF-BB (p = 0.041) and marginally significant for VEGF (p = 0.06) (Figure 2C).

## Discussion

This is the first description of the plasma profiles of a reasonably large panel of pro-inflammatory, anti-inflammatory and growth factors assessed simultaneously by multiplex technique among T1R and T2R patients compared to well-matched leprosy patients without reaction. The results indicate that CXCL10 is a promising biomarker of T1R, at least in BT patients, since those with T1R had a significant elevation of circulating CXCL10. This is the first observation of high levels of circulating CXCL10 in leprosy.

CXCL10, one of the IFN-γ inducible chemokines that recruits effector Th1 type cells to delayed type hypersensitivity sites, has been previously identified in tuberculoid lesions [8]. Although our study was not designed to investigate mechanisms, the higher levels of CXCL10 detected in T1R suggest its involvement in the T1R immunopathology probably by attracting Th1 type cells to the reactional inflammatory sites in the skin, (reviewed in [1]).

CXCL10 has been associated with Th1 type responses in several clinical systems recruiting CXCR3^+ ^effector lymphocytes from the peripheral blood into lupus erythematosus skin lesions [9], and into inflammatory lesions of the central nervous system [10]. CXCL10 has been proposed to be an immune marker of disease progression in HIV and tuberculosis patients [11] and was increased in sarcoidosis patients' serum. High levels of CXCL10 were found in the cerebrospinal fluid of multiple sclerosis patients' during intense inflammatory process [12-14]. CXCL10 has not been associated with inflammation of peripheral nerves but its possible involvement in the neuropathy associated with T1R deserves further study.

Another promising systemic marker of T1R is the inflammatory cytokine IL6, which promotes cell mediated immune reactions notably by stimulating IL17, and by inhibiting regulatory T cells. IL6 stimulates the synthesis of acute-phase proteins and is elevated in stressful inflammatory or infectious conditions [15]. Increased serum IL-6 has recently also been reported in T2R, and in lepromatous versus tuberculoid disease [16].

Greater IL7 was observed in LL patients with T2R compared to non-reacting LL patients. We did not observe greater circulating levels of IL7 in tuberculoid patients compared to lepromatous ones, unlike earlier skin biopsy studies which found greater IL7 mRNA expression in tuberculoid lesions versus lepromatous ones, and T2R lesions showed the weakest IL7 expression [17]. Different immunological activity in the central lymphoid system (reflected in the circulation) versus sites of inflammation in tissue may explain this. IL7 is a key regulator of B cell development and proliferation [18], and is essential for the survival of naïve and memory T cells, especially CD4 memory cells [19]. Elevated circulating levels of IL7 in T2R thus support a role for both B-cell and T-cell mediated mechanisms in this reaction.

CCL11 previously named eotaxin, a chemokine induced by interferon-γ and produced by monocytes, was identified as a potential plasma marker of T2R. Recently, greater plasma concentrations of CCL11 have been shown in Brazilian MB leprosy patients, when compared to non infected individuals [20]. CCL11 is a potent chemo attractant for eosinophils and probably other cell types such as Th2 lymphocytes, to inflammatory sites [21,22]. It is possible that CCL11 participates in attracting inflammatory cells to T2R sites.

Anti-inflammatory cytokines down-modulate inflammatory responses and reduce immunopathology. Among anti-inflammatory cytokines (IL4, IL10, IL13 and IL1RA) only IL10 showed marginally significant difference for T2R. This finding is in agreement with previous studies in plasma, IL10 mRNA in T2R skin lesions, and after in vitro stimulation of peripheral blood mononuclear cells of T2R patients [4,23-26].

Significant differences were observed between T2R and controls for the growth factors PDFG-BB and VEGF, both of them known to promote angiogenesis. This is an interesting finding as T2R are often associated with vasculitis [27,28].

While several studies indicate an important role for TNFα in reactional states, especially T2R [29-32], this finding has not always been confirmed [33,34]. Our results indicated that TNFα levels among reactional patients were not statistically different from the non-reactional patients. Previous studies with T2R patients have reported undetectable or low levels of TNFα [35] or have not found increased circulating TNFα [31,36]. This study evaluated levels of TNFα in plasma and used a multiplex bead assay, unlike most prior work that has used ELISA technology to assess cytokines in serum.

IL17, produced by pro-inflammatory TH17 lineage of effector CD4^+ ^T helper lymphocytes, induces the production of chemokines and antimicrobial peptides by tissue cells leading to the recruitment of neutrophils and inflammation [37]. In this study IL17 did not show significant difference between reactional and non reactional leprosy patients.

## Conclusion

This survey of circulating cytokines has identified elevations in CXCL10 and IL6 as promising markers for leprosy T1R. These findings are consistent with T1R cell-mediated immunolopathologic basis. IL7 and PDGF-BB represent potential markers of T2R. Additional studies may determine the sensitivity and specificity of these cytokines as leprosy reactions markers. The identification of T1R and T2R markers can contribute to their clinical diagnosis and for monitoring their resolution during treatment. In addition, these findings may provide new clues to the pathogenesis of leprosy reactions.

## Competing interests

The authors declare that they have no competing interests.

## Authors' contributions

MMS and DMS conceived of the study, carried out the immunoassay, participated in its design, coordination and drafted the manuscript; JGG and ALS were responsible for patients' diagnosis, recruitment, treatment and participated in the data analysis; MBC was in charge of the histopathological analysis; MLO participated in the design of the study; CMT participated in the design of the study, coordination and performed the statistical analysis. All authors read and approved the final manuscript.

## Pre-publication history

The pre-publication history for this paper can be accessed here:

http://www.biomedcentral.com/1471-2334/9/75/prepub
